# Association between dysphagia and tongue strength in patients with amyotrophic lateral sclerosis

**DOI:** 10.1016/j.bjorl.2020.10.015

**Published:** 2020-11-29

**Authors:** Alda Linhares de Freitas Borges, Leandro Castro Velasco, Hugo Valter Lisboa Ramos, Rui Imamura, Paula Martins Alves de Castro Roldão, Marcela Vieira Barbosa Petrillo, Claudiney Cândido Costa

**Affiliations:** a Centro de Reabilitação e Readaptação Dr. Henrique Santillo de Goiânia (CRER), Residência Médica em Otorrinolaringologia, Goiânia, GO, Brazil; b Hospital Servidor Público Municipal de São Paulo, São Paulo, SP, Brazil; c Universidade de São Paulo (USP), Faculdade de Medicina (FM), São Paulo, SP, Brazil; d Universidade Federal de São Paulo (Unifesp), São Paulo, SP, Brazil; e Universidade de São Paulo (USP), Faculdade de Medicina (FM), Hospital das Clínicas (HC), Serviço de Bucofaringolaringologia da Clínica Otorrinolaringológica, São Paulo, SP, Brazil; f Universidade Católica de Goiás, Goiânia, GO, Brazil; g Centro de Reabilitação e Readaptação Dr. Henrique Santillo de Goiânia (CRER), Equipe de Doenças Neuromusculares, Goiânia, GO, Brazil; h Centro de Reabilitação e Readaptação Dr. Henrique Santillo de Goiânia (CRER), Goiânia, GO, Brazil; i Pontifícia Universidade Católica de Goiás, Goiânia, GO, Brazil

**Keywords:** Deglutition disorders, Amyotrophic lateral sclerosis, Tongue

## Abstract

**Introduction:**

Amyotrophic lateral sclerosis is the most common motor neuron disease in adults despite it being rare. It is a neurodegenerative disease in which dysphagia is a common and debilitating symptom. Dysphagia can be assessed by complementary exams, such as fiberoptic endoscopic evaluation of swallowing and the tongue strength test, as this is one of the main muscles involved in swallowing.

**Objective:**

To compare the results of tongue strength and endurance measured by the Iowa oral performance instrument with the findings of the fiberoptic endoscopic evaluation of swallowing examination in patients affected by amyotrophic lateral sclerosis.

**Methods:**

Cross-sectional study, carried out in a tertiary hospital specialized in treatment and rehabilitation. Twenty-five patients diagnosed with amyotrophic lateral sclerosis underwent dysphagia questionnaires, fiberoptic endoscopic evaluation of swallowing examination and tongue strength and resistance test with the Iowa oral performance instrument to assess the presence of dysphagia.

**Results:**

Forty-eight percent of the sample had dysphagia at the fiberoptic endoscopic evaluation of swallowing and 76% had an altered tongue strength test. Ninety percent of patients with dysphagia had an average tongue pressure lower than 34.2 KPa. The tongue strength test showed sensitivity of 91.67% and specificity of 38.46% and accuracy of 64%. There was a statistically significant relationship between tongue strength and dysphagia and between tongue resistance and dysphagia.

**Conclusion:**

Tongue strength tests, such as the Iowa oral performance instrument, proved to be effective in assessing dysphagia. This result should encourage further research to facilitate the early diagnosis of dysphagia.

## Introduction

Amyotrophic lateral sclerosis (ALS) is the degenerative disease of the upper and/or lower motor neurons, without sensory or cognitive impairment, more commonly seen in adults.^1^, ^2^, ^3^ The disease has a progressive characteristic, with degeneration of the motor system at various levels–bulbar, cervical, thoracic, lumbar and appendicular.^1^

Many patients affected by ALS have difficulty swallowing as an initial symptom of the disease. The change in deglutition can affect any phase of swallowing and this change can develop into debilitating conditions.^4^ The early diagnosis of dysphagia is important for the prevention of malnutrition, dehydration and aspiration pneumonia pictures, in addition to allowing adequate treatment.^5^, ^6^, ^7^, ^8^

The objective assessment of swallowing can be carried out by complementary exams, namely swallowing videofluoroscopy (SVF) and fiberoptic endoscopic evaluation of swallowing (FEES), a highly accurate test and currently considered the gold standard by many authors.^5^, ^6^, ^9^, ^10^, ^11^, ^12^ These methods, however, are not used in most hospitals in Brazil, as they require trained professionals and expensive equipment.^5^, ^6^, ^7^, ^10^

In view of the limited availability of gold standard exams in the diagnosis of dysphagia, alternative exams with easier access have become a necessity. An alternative test option still under study is the measurement of tongue strength with portable pressure transducer tools, such as the Iowa oral performance instrument (IOPI), a reliable device, easy to transport and apply.^13^, ^14^, ^15^, ^16^ Tongue strength has shown to be a reliable indicator of bulbar involvement in several diseases, including ALS. Because it is one of the main muscles involved in the swallowing process, tongue weakness often leads to oral and/or pharyngeal dysphagia.^17^, ^18^ However, few studies to date have used objective, non-invasive quantitative tests to measure tongue strength and endurance in dysphagic patients.^13^, ^19^

The present study has as its primary objective to analyze the association between tongue strength and endurance as measured by the IOPI and the presence of dysphagia, having FEES as the gold standard exam, in patients affected by ALS. The secondary objective is to determine the sensitivity and specificity of IOPI in dysphagia detection.

## Methods

This was an observational and prospective study, carried out in a tertiary hospital specialized in the treatment and rehabilitation of patients with physical, intellectual, visual and hearing disabilities. The study was approved by the ethics and medical research committee (ethical committee protocol: 2,023,500).

Patients diagnosed with ALS confirmed according to the El Escorial criteria were studied, regardless of complaints of dysphagia, seen on an outpatient basis at the institution. Patients over 18 years of age who agreed to participate in the study were included. Patients with other pathologies associated with or distinct from ALS, which compromised swallowing, were excluded. Those who were not able to perform the tests proposed in the study were also excluded.

The study population consisted of 26 patients diagnosed with ALS undergoing outpatient followup at the institution. One of the participating patients was excluded due to the technical incapacity to perform the exam, totaling 25 participants of both genders, aged between 35 and 79 years and diagnosis attained between 10 months and 19.7 years. Demographic and clinical data were collected from patients, such as duration of disease, type of disease involvement (bulbar or appendicular), feeding route and BMI.

Subsequently, the tongue strength test was performed in all patients, using the IOPI device. A bulb filled with air, coupled to the portable pressure transducer was placed on the patient's hard palate, just behind the anterior incisor teeth, and the patient was asked to press the bulb with their tongue against the palate, as hard as possible, for approximately 2 s. The peak pressure was expressed in kilopascals (KPa). Three tongue strength measurements were performed, with a 30 s interval between them. The highest value of peak isometric pressure between the three measurements was considered the maximum isometric pressure (MIP). The measurement of tongue endurance (IOPI-E) was also performed by quantifying the time (duration) that the patient was able to maintain 50% of their maximum pressure. This test was performed only once on each patient and always by the same examiner.

The participants were subsequently submitted to FEES using a flexible fiberscope, measuring 3.2 mm in diameter, brand Machida, coupled to a non-portable xenon light source, brand Ecleris, and the camera, model Opitice Pro HD 2, brand GoPro. The images were stored on a computer with an image scanner, model Infoco 2 lite, brand Infoco, and on a DVD/CD recorder.

The fiberscope was introduced through the nostril without administration of a vasoconstrictor or anesthetic to the nasal mucosa, so as not to interfere with pharyngolaryngeal sensitivity. The FEES exam routine followed the protocol described by Langmore.^20^ To evaluate swallowing, samples stained with aniline blue were used in the following consistencies: liquid (3, 5 and 10 mL), thickened liquid (3, 5 and 10 mL), semi-solid (3, 5 and 10 mL) and solid (¼ of a cracker). The endoscope was positioned just above the epiglottis. After deglutition, the endoscope tip was placed close to the vocal folds to assess the presence of residues, penetration and aspiration. The degree of dysphagia was divided into mild, moderate and severe, according to the classification by Macedo et al.^21^ The result was categorized as normal (absence of dysphagia) and altered (presence of mild, moderate or severe dysphagia). All exams were performed in an outpatient setting, with the patient in the sitting position.

The evaluations of tongue strength and endurance and the FEES were performed sequentially on the same day. The FEES was monitored by another speech therapist specialized in dysphagia and an otorhinolaryngologist, and both examiners had no participation or knowledge of the tongue assessment results.

The results were analyzed using the Windows Statistical Package for Social Science (SPSS) program (version 21.0). The association of aspiration with tongue strength and endurance was assessed using Mann–Whitney’s test and Fisher's test, respectively. The association of aspiration with age was assessed by Student's *t*-test and the association of aspiration with gender was assessed by Pearson's Chi–square test. The Kolmogorov–Smirnov test was applied to assess the normality of the quantitative variables, and variables with *p* values > 0.05 were considered to have a normal distribution. Spearman's correlation coefficient was applied to verify the correlation between two quantitative variables, considering r = 0 a null correlation; 0 > r < 0.3, a weak correlation; 0.3 ≥ r < 0.6, a moderate correlation; 0.6 ≥ r < 0.9, a strong correlation; 0.9 ≥ r < 1.0, a very strong correlation and r = 1, a perfect correlation. Sensitivity, specificity, Positive Predictive Value (PPV), Negative Predictive Value (NPV) and MIP accuracy were calculated to detect dysphagia using the FEES as the gold standard test. A 95% confidence level was considered for all tests, that is, a *p* value < 0.05 was considered significant.

## Results

Of the 39 patients with ALS undergoing outpatient followup, 26 agreed to participate and were included in the study. One patient was excluded due to the difficulty of undergoing the FEES exam. Therefore, 25 patients with ALS were analyzed, whose epidemiological data are summarized in Table 1, Table 2. All patients had oral feeding as the main nutrition route.

**Table 1:** Descriptive analysis of Amyotrophic Lateral Sclerosis cases according to the presence of dysphagia.

Variables	Dysphagia		*p* value
	Yes	No	
Number of cases	13	12	
Mean age (SD)	57.08 (13.43)	52.00 (8.64)	0.268^a^

Gender			
Male	8	7	0.688^b^
Female	4	6	
Median time of diagnosis in months (IQR)	16 (9.50–45.75)	14.00 (11.00–31.00)	0.849^c^

SD, Standard Deviation; IQR, Interquartile Range.

a Student’s *t*-test.

b Fisher’s exact test.

c Mann–Whitney test.

**Table 2:** Descriptive analysis of cases of amyotrophic lateral sclerosis according to the type of disease involvement.

Variables	Type		*p* value
	Appendicular	Bulbar	
Number of cases	20	5	
Mean age (SD)	54.55 (12.03)	54.00 (8.49)	0.925^a^

Gender			
Male	14	1	0.121^b^
Female	4	4	
Median time of diagnosis in months (IQR)	18.50 (13.00–45.75)	11.00 (9.00–17.50)	0.071^c^

SD, Standard Deviation; IQR, Interquartile Range.

a Student’s *t*-test.

b Fisher’s exact test.

c Mann–Whitney test.

Twenty (80%) of the patients had a diagnosis of appendicular ALS; of these, 11 (55%) had altered FEES results and 14 (70%) had altered tongue strength (Table 2). Five (20%) of the patients had bulbar ALS, of which 2 (40%) had altered FEES results and 100% had reduced tongue strength (Table 3). Weight loss was observed in 14 (56%) patients, and only 9 (36%) had a BMI ≥ 25.

**Table 3:** Association between tongue strength and tongue endurance with Fiberoptic Endoscopic Evaluation of Swallowing (FEES).

		Dysphagia				*p* value*
		Normal (n = 13)		Altered (n = 12)		
		N	%	N	%	
IOPI-E	Normal (> 10 seg)	12	92.3%	6	50.0%	0.03^a^
	Altered (≤ 10 seg)	1	7.7%	6	50.0%	
MIP	Mean (DP)	35.7 (16.4)		12.2 (11.1)		0.001^b^
	Median (min–max)	31 (4–60)		9 (2–43)		

SD, Standard Deviation; min, minimum value; max, maximum value; MIP, Maximum Isometric Pressure; IOPI R, (Iowa Oral Performance Instrument), tongue endurance.

* *p* < 0.05.

a Fisher’s exact test.

b Mann–Whitney test.

Age, gender and time since diagnosis did not show a statistically significant association with the presence of dysphagia at the FEES (*p* = 0.26, *p* = 0.68 and *p* = 0.85, respectively) (Table 1). There was also no statistically significant association between these variables and the type of disease, appendicular and bulbar (*p* = 0.92, *p* = 0.12 and *p* = 0.070 respectively) (Table 2).

Of the analyzed patients, 12 (48%) had dysphagia at the FEES: 4 (33.3%) mild, 5 (41.6%) moderate and 3 (25%) severe cases.

Of the 22 patients with exclusive oral feeding, 5 (23%) showed penetration during the swallowing of some food consistencies and 2 (9%) had aspiration during the exam.

The tongue strength test was found to be altered in 19 (76%) of the patients and the endurance measurement in 7 (24%). Ninety percent of the patients with dysphagia had a MIP < 34.2 kPa. There was a statistically significant association between MIP and dysphagia (*p* = 0.001). There was also a statistically significant association between IOPI -E and dysphagia (*p* = 0.030).

When assessing the correlation of the dysphagia degree with the maximum pressure measured and tongue endurance, there was a strong negative correlation between the degree of dysphagia and the maximum pressure measured (*p* = 0.001; r = −0.611), that is, the higher the degree of dysphagia, the lower the measured pressure value. No correlation was observed between the degree of dysphagia and tongue endurance (Table 4).

**Table 4:** Correlation between the degree of dysphagia with the highest pressure measured and tongue endurance.

	Degree of dysphagia	
	*p*	r
Highest measured pressure	0.001	−0.611
Tongue endurance	0.319	−0.208

Statistical test: Spearman’s Correlation Coefficient.

We calculated the performance of tongue strength in detecting dysphagia using the FEES as a reference test (Table 5). MIP had a sensitivity of 91.67% and specificity of 38.46%. The test's accuracy was 64%, with a positive predictive value of 57.89% and a negative predictive value of 88.33%. The estimated prevalence of dysphagia according to the test was 76% (95% CI: 56.57–88.50) and by FEES, 48% (95% CI: 30.03–66.50).

**Table 5:** Sensitivity, specificity, accuracy, positive and negative predictive values of the tongue pressure test in relation to the Fiberoptic Endoscopic Evaluation of Swallowing.

IOPI	Sensitivity % (95%CI)	Specificity % (95%CI)	PPV % (95%CI)	NPV % (95%CI)	Accuracy % (95%CI)
MIP	91.67	38.46	57.89	88.33	64

IOPI, Iowa Oral Performance Instrument; MIP, Maximum Isometric Pressure; CI, Confidence Interval; PPV, Positive Predictive Value; VPN, Negative Predictive Value.

## Discussion

This study sought to assess the findings of FEES and compare them with tongue strength and endurance in patients with ALS, a disease with highly variable behavior, and rapid progression to severe dysphagia. Our study showed a higher incidence in men and a higher prevalence of the appendicular type (80% of the sample), in addition to a high incidence of dysphagia in patients with this disease, corroborating the findings of other studies evaluating the disease.^1^, ^2^, ^3^

Previous studies evaluating other neurological pathologies that affect swallowing disorders have observed a positive association between tongue strength and dysphagia.^14^, ^15^ In our study, dysphagic patients had significantly reduced tongue strength and endurance, when compared to patients with preserved swallowing. Hiraoka et al.,^22^ in a study similar to ours, observed that the maximum tongue pressure was significantly lower in patients with swallowing alterations.

The tongue strength test showed high sensitivity (91.67%), demonstrating that this test is capable of detecting most patients with dysphagia, constituting a plausible alternative for an early and accessible assessment of this alteration. However, the specificity (38.46%) showed to be a limitation of the test, since patients without dysphagia may have altered IOPI results. This reduced specificity is probably related to the technical difficulty in performing the exam, due to the lack of coordination that many of the patients with neuromuscular diseases have, in addition to the difficulty in understanding, observed in some stages of neurodegenerative diseases. This increase in the number of false positives would lead to an increase in unnecessary complementary tests and, thus, to higher costs with health services. Despite this reduced specificity, the tongue strength test seems adequate for screening the early stages of neuromuscular diseases, such as ALS. It is known that the early diagnosis of swallowing disorders can prevent some complications related to disease progression, and that increasingly accessible tests are essential.

Robinovitch et al.^16^ analyzed 6 patients with no swallowing alterations and two with dysphagia using a computer-assisted tongue strength measurement system and observed that dysphagic patients have reduced tongue strength. Stierwalt et al evaluated tongue strength in 35 patients with dysphagia using the IOPI, comparing them with a control group with the same epidemiological characteristics, and demonstrated that the dysphagic patients had significantly reduced tongue strength and that the IOPI was able to quantify this difference.^23^

Easterling et al.,^24^ in a study with ALS patients diagnosed 24 months before, determined their isometric strength of the tongue using the IOPI, observing an average pressure of 35.89 kPa in the group with bulbar symptoms and 41.51 kPa in the group with appendicular symptoms. In this study, the ALS group with bulbar symptoms had an average tongue strength of 12.64 kPa and the group with the appendicular symptoms, 23.08 kPa. This difference is probably due to the disease duration, which ranged from 10 months to 19.6 years, with an average of 13.98 months for bulbar ALS and 38.45 months for appendicular ALS.

Onesti et al.^25^ evaluated 145 patients with ALS, 39% with the bulbar and 61% with the appendicular type and found a prevalence of 58.6% of dysphagia in patients with an average time of disease diagnosis of 15.8 ± 12.7 months. The prevalence of the presentation forms is consistent with those found in our study, in which 40% of the sample had bulbar and 60% had appendicular ALS. The prevalence of dysphagia, however, was lower in our study (48%), probably due to the difference in sample size and due to the used classification; in the abovementioned study, the Aspiration Penetration scale was used to define swallowing impairment, which is a non-gradual scale. The Brazilian study by D′Ottaviano et al.,^7^ showed a prevalence of 100% of dysphagia in patients with ALS.

Dysphagia is undoubtedly one of the most debilitating problems of ALS. Its prevalence is high in patients with this disease and it needs an early diagnosis for patients to attain a better quality of life. Low-cost tests that are easy to apply are increasingly more necessary, guaranteeing greater accessibility to patients with disorders that present with dysphagia, such as ALS and many other neurodegenerative diseases. Despite the need for further studies, mainly in patients with impaired oral phase, the tongue strength test can be a screening option that becomes increasingly viable to be used in the investigation of this symptom.

## Conclusion

Tongue strength and endurance measures are significantly associated with dysphagia in ALS patients. The IOPI, test of tongue strength and endurance, has good sensitivity, but low specificity for detecting dysphagia. The findings of this study provides an alternative screening test for dysphagia. This result should encourage further research with a larger sample to consolidate these findings, since the low cost and easy applicability of the IOPI may help in the rapid and early diagnosis of dysphagia in these patients.

## Conflicts of interest

The authors declare no conflicts of interest.
